# Admission prevention in COPD: non-pharmacological management

**DOI:** 10.1186/1741-7015-11-247

**Published:** 2013-11-20

**Authors:** Eui-Sik Suh, Swapna Mandal, Nicholas Hart

**Affiliations:** 1 Lane Fox Clinical Respiratory Physiology Research Centre, Guy’s and St Thomas’ NHS Foundation Trust, London, UK; 2 Division of Asthma, Allergy and Lung Biology, King’s College London, London, UK; 3 Lane Fox Respiratory Unit, Guy’s and St Thomas’ NHS Foundation Trust, London, UK

## Abstract

Exacerbations of chronic obstructive pulmonary disease (COPD) are one of the commonest causes of hospital admission in Europe, Australasia, and North America. These adverse events have a large effect on the health status of the patients and impose a heavy burden on healthcare systems. While we acknowledge the contribution of pharmacotherapies to exacerbation prevention, our interpretation of the data is that exacerbations continue to be a major burden to individuals and healthcare systems, therefore, there remains great scope for other therapies to influence exacerbation frequency and preservation of quality of life. In this review, the benefits and limitations of pulmonary rehabilitation, non-invasive ventilation, smoking cessation, and long-term oxygen therapy are discussed. In addition, supported discharge, advanced care coordination, and telehealth programs to improve clinical outcome are reviewed as future directions for the management of COPD.

Please see related article: http://www.biomedcentral.com/1741-7015/11/181.

## Financial and human cost

Management of chronic obstructive pulmonary disease (COPD) is a worldwide challenge. It has a prevalence of 1.5% in the UK [1] and 5.1% in the USA [2], while in China, which has about one-third of the world’s smokers, the prevalence of COPD in patients aged over 40 years is estimated at 8.2% [3]. Current predictions estimate an annual COPD mortality rate in China of over 2 million by 2033 [4]. As expected, COPD imposes a substantial economic burden on healthcare systems. Data from the USA showed that in 1 year, COPD caused 1.5 million emergency department (ED) attendances, 726,000 hospitalizations, and 119,000 deaths [5]. Direct costs of COPD have been estimated at $29.5 billion, with indirect costs of $20.4 billion [6]. Studies in the UK have estimated an annual direct cost of treatment per patient of £819 [7]. Given the heterogeneity of COPD, it is not surprising that acute exacerbations of COPD display a broad range of phenotypes, which can be categorized by their clinical, physiological, radiological, and etiological features [8]. These are discussed in detail in an earlier review on this subject in this journal [9]. Although exacerbation phenotyping can facilitate the targeting of treatments to individual patients, the severity of the exacerbation determines the urgency and location of treatment, and this pragmatic classification is in widespread use (Table 1) [10]. Up to 50% of exacerbations are mild and may go unreported, with 40 to 45% being classified moderate and less than 10% as severe [10].

**Table 1:** **Classification of exacerbation severity**[10]

**Level**	**Description**
Mild	An increase in respiratory symptoms that can be controlled by the patient with an increase in the usual medication
Moderate	Requires treatment with systemic steroids and/or antibiotics
Severe	Requires hospitalization or a visit to the ED

An acute exacerbation of COPD has detrimental effects on lung function [11-14], health-related quality of life (HRQL) [15-17] and exercise capacity [18]. Several studies have shown high mortality rates for patients with COPD who are hospitalized with an acute exacerbation [19-23]. The SUPPORT study reported an in-hospital mortality rate of 11% in patients with COPD admitted with hypercapnic respiratory failure, and 2-year mortality was 49% [23]. Soler-Cataluna *et al.* demonstrated, in a large Spanish cohort of patients with COPD, the relationship between exacerbation frequency and mortality [19]. Whereas exacerbation-free patients had a survival rate of 80%, patients with three or more exacerbations per year had a 5-year survival rate of only 30%. A recent systematic review of 37 studies involving 189,772 hospitalized patients with COPD reported 12 factors (Table 2) associated with short-term mortality (90 days after hospital discharge) and 9 factors associated with long-term mortality (2 years after hospital discharge) (Table 2) [24].

**Table 2:** **Predictors of early and late mortality in hospitalized patients with acute exacerbation of chronic obstructive pulmonary disease (adapted from Singanayagam *et al*.**[24]**)**

**Predictors of short-term mortality (up to 90 days after hospitalization)**	**Predictors of long-term mortality (up to 2 years after hospitalization)**
Age	Age
Male sex	Low body mass index
Low body mass index	Cardiac failure
Cardiac failure	Diabetes mellitus
Chronic renal failure	Ischemic heart disease
Confusion	Malignancy
Long-term oxygen therapy	FEV_1_
Lower limb edema	Long-term oxygen therapy
GOLD stage 4 disease	P_a_O_2_ on admission
Cor pulmonale	
Acidemia	
Raised plasma troponin level	

*Abbreviations*: *GOLD* Global Initiative for Obstructive Lung Disease, *FEV*_*1*_ forced expiratory volume in 1 second, *P*_*a*_*O*_*2*_ partial pressure of oxygen in arterial blood.

## Hospital admission and re-admission

Intolerable dyspnea is the major cause of hospital admission during an exacerbation of COPD [25]. This event is common, with hospitalization due to dyspnea accounting for one in eight hospital admissions in the UK and one in four admissions in Canada [26]. In the UK, such patients occupy a hospital bed for a median of 5 days [27], with a 20% re-admission rate within 28 days, and up to a third of patients re-admitted within 3 months [27,28]. These data differ from other parts of Europe and from the USA, where 30-day re-admission rate is estimated at 10.9% and 8.1%, respectively [29]. Despite these differences, this is clearly a burden to both healthcare systems and patients, and as of 2011, the UK National Health Service has limited the reimbursement to acute hospitals for patients who are re-admitted within 30 days. Similar key performance targets have been imposed in the USA [30]. Although inhaled and oral drug preparations play an important part in exacerbation prevention, the reduction with pharmacotherapy of severe exacerbations requiring hospitalization is limited. Non-pharmacological management therefore has an important role in the management both of patients with stable disease at risk of exacerbation and of those who are in the immediate recovery phase following an acute exacerbation.

## Non-pharmacological management

### Pulmonary rehabilitation

#### Physiological principles

Pulmonary rehabilitation (PR) is ‘an evidence-based, multidisciplinary and comprehensive intervention for patients with COPD that is designed to reduce symptoms, optimize functional status, increase patient participation and reduce healthcare costs through stabilizing or reversing systemic manifestations of the disease’ [31]. Exercise training is a major component of PR, and aims to modify skeletal muscle function to enhance exercise capacity [32,33]. Improved skeletal muscle performance and subsequent reduced lactate production throughout exercise enhances the relationship between respiratory muscle load and respiratory muscle capacity. This is achieved through modifications in breathing pattern, in the context of airflow limitation with optimization of pulmonary mechanics, which reduces exertion-related dyspnea, with a resultant improvement in exercise capacity leading to further improvement in skeletal muscle performance [34,35].

In addition to impaired exercise capacity and disuse in the stable state, several other mechanisms have been implicated in the muscle wasting and weakness associated with exacerbation [36]. Systemic inflammation, confirmed by an elevation in serum interleukin (IL)-6 and IL-8 levels during acute illness, has been shown to have an inverse relationship with quadriceps muscle strength [37]. In addition, oxidative stress is prominent in the peripheral skeletal muscle of patients during an acute exacerbation, which adversely affects mitochondrial function and the contractile properties of the skeletal muscle [38]. Furthermore, blood gas abnormalities affect skeletal muscle function, with hypoxemia being associated with muscle weakness, inhibition of protein synthesis, and activation of proteolysis [39], while hypercapnic acidosis worsens skeletal muscle fatigability [40] and the endurance properties of the diaphragm [41]. Muscle wasting as a consequence of systemic corticosteroid treatment through the inhibition of protein synthesis, downregulation of the anabolic insulin-like growth factor-1 pathway, and activation of catabolic pathways, combined with appetite suppression and reduced dietary intake due to systemic inflammation during acute illness, drives the energy imbalance between supply and demand [42].

Although exercise training plays an important role in improving patient outcomes in COPD, other components contribute significantly to the benefits of PR. PR also addresses the nutritional deficits that are common in stable COPD and during acute exacerbations [43]. Low body mass index is associated with poor prognosis in patients with COPD [44], and caloric supplementation may help to maintain or restore body weight and fat mass, and ensure adequate protein intake. The patient education component of PR is aimed at self-management and enhanced autonomy, in order to encourage early self-identification and treatment of exacerbations. Education programs may also include breathing strategies to control dyspnea as well as bronchial hygiene techniques [43]. Psychosocial support may help to address the anxiety, depression, and other mental health problems that are often associated with chronic respiratory disease [45,46].

#### Pulmonary rehabilitation delivered in the post-acute exacerbation recovery stage

Although well established as part of chronic care in stable COPD [43,47], there is mounting evidence for the utility of PR in the early recovery period following an exacerbation [48]. Furthermore, data support the role of PR in preventing exacerbations and in reducing acute healthcare utilization, including unscheduled physician visits, ED attendances, and hospital admissions [49]. There are specific features of acute exacerbations that make them an important target for PR. Skeletal muscle dysfunction is evident, with a decline in quadriceps muscle strength of 5% between day 3 and 8 of hospital admission [37]. In the absence of any intervention, quadriceps force continues to decline for up to 3 months after hospital discharge [50]. Immobility and reduced physical activity are major contributors to muscle wasting and weakness, with hospitalized patients spending less than 10 minutes per day walking [51]. Furthermore, these patients remain inactive for up to 1 month after discharge compared with patients with stable COPD and similar disease severity.

Patients are at high risk of re-exacerbation and re-admission in the early recovery phase. Therefore, there is a potential role for an intervention in the post-exacerbation period after an acute episode to reduce the re-admission risk. A recent Cochrane systematic review of five randomized controlled trials (RCTs) of early PR post-acute exacerbation [48] concluded that there was a significant reduction in hospital admissions in patients enrolled in PR programs following an exacerbation (odds ratio 0.22, 95% confidence interval 0.08 to 0.58). More importantly, these data showed that only four patients need to receive PR in the post-acute phase in order to prevent one re-admission, with an overall reduction in mortality observed also (OR 0.28; CI 0.10 to 0.84). There were no serious adverse events in any of the five studies reviewed. Although Eaton *et al.* showed only a trend towards a reduction in 90-day re-admission in the PR group compared with the usual care (UC) group (23% versus 32%, respectively), the adherence to the PR program was only 40% [52]. However, in the trial of Seymour *et al.*, the effect of early outpatient PR on 3-month re-admission rate was investigated in patients enrolled within 1 week of hospital discharge [50]. The intervention group in this study received 16 exercise training sessions over a 3 month period rather than the standard PR approach of 2 sessions per week for 8 weeks. This ensured that the effect of the treatment was tested, whereas the results of the trial by Eaton *et al.*[52] were, in part, a consequence of the failure of delivery of the treatment rather than necessarily a failure of the treatment itself. In the Seymour study, re-admission rate at 3 months was lower in the early PR group compared with the UC group (7% versus 33%, respectively; *P* = 0.02) [50]. Interestingly, Seymour *et al.* reported that the rate of ED attendances not requiring admission were similar between the two groups, but the rate of hospital attendance of any type was lower in the early PR group (27% versus 57%; *P* = 0.02). The post-discharge frequency of exacerbations was lower in the early PR group (0.27 versus 1.1; *P* < 0.01). Although informative, these trials were limited by a relatively short-term follow-up period. By contrast, Ko *et al.* investigated the effect on healthcare utilization at 12 months of an 8-week program of supervised outpatient PR in patients enrolled up to 3 weeks following hospital discharge [53]. Although the PR group showed improvement in health status at 3 and 6 months, this effect did not persist at 12 months, and there was no reduction in healthcare utilization at 12 months. Similarly, Puhan *et al.* reported, albeit in an underpowered study, that early PR failed to improve exacerbation rate at 18 months [54]. This is not surprising as the patients enrolled in such trials have severe and very severe COPD, and the interventions that are applied are unlikely to have effect on long-term benefit as the disease process progresses. Despite this, the short-term gains to the patient and acute healthcare providers are clear. In the future, we may target these patients during the exacerbation as inpatients. Acknowledging that these patients have very high levels of dyspnea during this period, which prevents exercise, novel use of technologies that accommodate for or modify dyspnea, such as neuromuscular electrical stimulation [55] and non-invasive ventilation [56], have been used as adjuncts to exercise training in pilot studies, but further work is required.

#### Pulmonary rehabilitation delivered in the stable state

Although uncontrolled cohort studies have found that PR reduces hospitalization frequency [57,58] and hospital bed days [57-60], RCTs of PR in patients with stable COPD have not shown such consistent results. Griffith *et al.* reported that despite fewer hospital bed days, there was no reduction in hospitalization frequency in the PR group [61]. Guell *et al.* found a reduction in hospital admissions over a 2-year period [62]; however, other studies have failed to show a reduction admission frequency and hospital bed days [63,64]. These contrasting data highlight the differing phenotypes of COPD, based on exacerbation frequency, and the requirement for clinicians to develop strategies to target the timing of PR based on the phenotype rather than on the current clinical state of the patient at the time of starting PR.

#### Non-invasive ventilation

Non-invasive ventilation (NIV) is well established as the treatment of choice for patients with COPD with acute decompensated hypercapnic respiratory failure (AHRF) who fail to respond to standard medical therapy [65,66]. Importantly, and in addition to a reduction in mortality, hospital length of stay is reduced compared with standard treatment. Although NIV remains controversial as a domiciliary treatment to reduce hospital admission and improve survival in patients with COPD with stable chronic respiratory failure, the physiological mechanisms by which long-term NIV results in clinical improvement in patients with severe COPD and hypercapnic respiratory failure are well-described [67]. Indeed, Nickol *et al.* showed that 3 months of NIV enhanced gas exchange through alterations in pulmonary mechanics (shown as reduced gas trapping), and also increased ventilatory sensitivity to carbon dioxide. However, there was limited effect on non-volitional muscle strength [68]. Clinical manifestations of these physiological changes are reflected as reduced dyspnea and improved HRQL, and it is hypothesized that there will be an associated reduction in acute exacerbations and hospitalization, with a potential for improved survival. However, trial data are as yet inconclusive for this high-risk group of patients with severe COPD.

An observational study from Tuggey *et al.* showed that, following initiation of domiciliary NIV in a cohort of patients with COPD who were prone to recurrent admissions, there was a significant reduction in total hospital days and days spent in the intensive care unit. This was, not unexpectedly, associated with substantial cost savings [69]. This is in contrast to several RCTs that have failed to show a convincing benefit in terms of acute healthcare utilization. In the trial by Casanova *et al.*, there was no difference in survival between patients with stable COPD randomized to NIV or UC [70], although the proportion of patients who required hospital admission at 3 months was reduced in the intervention group (5% versus 15%; *P* < 0.05, respectively). Unusually, ventilator set-up in this trial was aimed at reducing accessory muscle use and reducing dyspnea, which explains, in part, the low inspiratory positive airway pressure (IPAP) of 12 cm H_2_O applied. Clini *et al.* randomized 90 patients to long-term oxygen therapy (LTOT) alone or home NIV (IPAP 14 cm H_2_O) with LTOT, as part of a multicentre trial [71]. Adherence to NIV was high in this study at 9 hours per day, but there was only a trend to a reduction in hospital admission comparing admission rate before and after enrolment (45% decrease in hospital admissions in the intervention group versus 27% increase in the UC group). More recently, McEvoy *et al.* found a significant improvement in survival in a combined NIV and LTOT group in both intention-to-treat and per-protocol (>4 hours NIV use per night) analyses (HR 0.63, 95% CI 0.40 to 0.99, *P* = 0.045 and HR 0.57, CI 0.33 to 0.96, *P* = 0.036, respectively) [72]. This was achieved with an adherence of 4.3 hours per night. Despite this beneficial effect, NIV in addition to LTOT treatment conferred no benefit in terms of HRQL or hospital admission, albeit the IPAP in this trial was again low, at 12.9 cm H_2_O.

Patients with COPD are at greatest risk of death and re-admission immediately after an episode of AHRF. Indeed, the reported re-admission rate is 79.9% with a 1-year mortality rate of 49.1% [73]. Two recent trials have focused on this high-risk group [74,75]. In the trial by Cheung *et al.*, patients who had required NIV for AHRF were randomized to domiciliary nocturnal NIV or continuous positive airway pressure (CPAP) of 5 cm H_2_O. The intervention was shown to have a lower rate of recurrent AHRF compared with the control group (38.5% versus 60.2%; *P* = 0.039, respectively) and a longer median time to first re-admission (71 days versus 56 days; *P* = 0.048, respectively) [74]. CPAP as an appropriate control arm in patients with COPD is interesting. The methodological aim was to balance the possible negative physiological effects of CPAP in patients with severe COPD against the concerns about using a control group that were not exposed to a mask interface. The use of interface with minimal pressure delivery allowed testing of the hypothesis that NIV is beneficial in COPD patients with post-acute hypercapnic respiratory failure. In a separate trial, Funk *et al.* enrolled patients who had required NIV for AHRF, but randomized the patients, after a run-in period on NIV of 6 months, to either continuation or withdrawal of NIV. The primary endpoint was escalation of ventilation. They found that the rate of ventilation escalation was lower in the NIV continuation group compared with the withdrawal group (15% versus 77%; *P* = 0.0048, respectively) [75]. These studies suggest a benefit of using domiciliary NIV in patients who are recovering from a recent acute exacerbation complicated by acute hypercapnic respiratory failure.

At present, there is controversy about the use of domiciliary NIV, and there are currently no widely accepted criteria for commencing domiciliary NIV in stable COPD, despite the practice being widespread [76]. The available data indicate that patient selection is important. Specifically, the patients most likely to benefit from long-term domiciliary NIV are those who exhibit symptomatic chronic hypercapnic respiratory failure and those with severe episodes of acute exacerbation requiring acute NIV during hospital admission [77]. Because the prognosis is better for patients who have hypercapnia that is reversible during the post-exacerbation recovery phase [78,79], it is important to target long-term NIV to patients who remain hypercapnic following their acute episode, as shown by the studies of Cheung *et al.* and Funk *et al.*[74,75]. Preliminary screening data in 25 patients from a UK RCT of post-exacerbation domiciliary NIV suggest a prevalence of persistent severe hypercapnia (arterial partial pressure of carbon dioxide > 7 kPa 2 weeks after an episode of AHRF) of over 40% [80]. However, further studies are needed to elucidate the trajectory of hypercapnia in a large cohort of patients with COPD treated with acute NIV.

Two RCTs are ongoing in the UK [81] and the Netherlands [82] to establish the effect of domiciliary NIV in reducing mortality and hospital admission for patients with COPD who are hypercapnic. The UK trial is focused on patients following an acute hospital admission requiring NIV, and the trial from the Netherlands is focused on patients with stable COPD who are hypercapnic. There are, however, several challenges in conducting such studies. Firstly, the absence of a true placebo for NIV makes it difficult to have a robust control group for comparison. Most studies to date have compared NIV with UC, with or without LTOT [70-72,75], but a limitation of this approach is that it does not take into account the placebo effects of being given a mask interface. Cheung *et al.* attempted to address this by administering nasal CPAP at 5 cm H_2_O to patients in the control group [74]. However, as the authors acknowledged, the possibility remained that the CPAP had a beneficial physiological effect on the control group, and could not therefore be considered to be a true placebo [83,84]. Secondly, the interpretation of the potential benefits of NIV are hampered by relatively short follow-up periods in the trials published to date; only two studies [71,72] have followed patients up for 2 years or more. Clearly, as patients established on domiciliary NIV are likely to remain on it for several years, it would seem advantageous for future studies to assess its benefits over the longer term.

### Smoking cessation

Smoking cessation is one of the few interventions shown to reduce mortality in patients with COPD. However, there are relatively few data showing the benefits of smoking cessation in reducing exacerbations. In the Lung Health Study, there was no significant difference in the risk of hospital admission between current smokers and ex-smokers [85]. Furthermore, Kessler *et al.* reported that smoking status had no effect on hospitalization risk [86], and Garcia-Aymerich *et al.* showed that current smoking was associated with a reduced risk of hospitalization in a small cohort of patients with COPD [87], suggesting that patients with very advanced disease and high risk of hospital admission quit tobacco consumption as a result of their significant symptom load. By contrast, Godtfredson *et al.* reported that, in a large prospective population study in Denmark, previous smokers had a lower risk of hospitalization for COPD (HR 0.57, 95% CI 0.73 to 1.18) compared with current smokers [88]. Interestingly, tobacco consumption (low versus high) had no effect on hospital admission. This study is supported by a population study by Au *et al.*, who reported a reduced risk of COPD exacerbations in ex-smokers compared with current smokers when adjusted for comorbidity, markers of COPD severity, and socioeconomic status (adjusted HR 0.78, 95% CI 0.75 to 0.87) [89]. Importantly, the duration of smoking abstinence significantly influenced the magnitude of the reduced exacerbation risk.

### Long-term oxygen therapy

Although LTOT is well established as a treatment to improve survival in patients with COPD and hypoxemia, there was no effect on exacerbation or hospitalization rates in early studies [90,91]. The benefits of LTOT in reducing acute healthcare utilization have been shown in the EFRAM cohort, with appropriate LTOT utilization being associated with lower risk of admission [87]. Further evidence was given by Ringbaek *et al.*, who showed in a Danish COPD cohort that LTOT reduced admission rates and hospital days by 23.8% and 31.2%, respectively [92].

### Risk stratification and physiological monitoring

Early recognition and treatment of exacerbations, and timely detection of treatment failure during an exacerbation are key factors that may reduce in hospital admissions, facilitate early discharge, and avoid re-admissions. The development of clinical tools to achieve this should be a priority for COPD research. Although there has been a considerable focus on molecular biomarkers, the predictive value of the data has been disappointing [93]. However, more encouraging data have shown that fibrinogen levels, as a biomarker of severity of systemic inflammation, combined with forced expiratory volume in 1 second (FEV_1_) predicted moderate to severe exacerbations in the following year [94].

Despite the limited clinical usefulness of the molecular biomarkers, basic and advanced physiological measurements have been shown to have increased utility in monitoring the course of COPD. Stevenson *et al.* showed that inspiratory capacity, as a marker of dynamic hyperinflation, changed significantly during the course of recovery from an exacerbation, while the impedance of the respiratory system, as measured by impulse oscillometry, was unchanged [95]. Murphy *et al.* investigated the use of a novel technique using electromyography of the second intercostal space parasternal muscle as an advanced physiological biomarker of neural respiratory drive in patients with COPD admitted to hospital with an exacerbation. Indices of neural respiratory drive were shown to be superior to standard bedside clinical measures and spirometry in detecting clinical deterioration [96]. Furthermore, when the neural respiratory drive between admission and discharge were compared, this physiological biomarker had the sensitivity and specificity to identify those patients who were re-admitted within 14 days. Advanced physiological technology that monitors the clinical status of the patient during hospital admission will not only identify treatment failure early but will also allow risk stratification for early re-admission. Furthermore, the technology has the potential to be used as part of a home telehealthcare program.

### Supported discharge and telehealth programs

Supported discharge and hospital-at-home programs have been introduced to improve the quality of life of patients by reducing hospital attendance and admission. This has potential benefits both for the patient and for reducing the expenditure within acute healthcare organizations. Studies have shown that in patients with uncomplicated acute exacerbations, early supported discharge is safe, and reduces length of stay without an increased re-admission rate, which was an initial clinical concern [97,98]. However, a meta-analysis of hospital at-home programs, as an alternative to continued hospitalization, concluded from eight trials that there was only a small benefit in terms of re-admission risk (risk ratio 0.76, 95% CI 0.59 to 0.99, *P* = 0.006) [99]. Furthermore, only a third of all patients were eligible for enrolment in the program, and there was no significant reduction in mortality (RR 0.65, 95% CI 0.4 to 1.04). Importantly, there was also no evidence of a cost saving.

With advances in information technology, advanced care coordination telehealth systems have been developed. These aim to facilitate transfer of clinical data about the patient through telecommunication networks. These data are reviewed remotely by a trained healthcare professional, who provides advice on the basis of the transmitted data [100]. In patients with COPD, such systems are aimed at early recognition and treatment of exacerbations in order to reduce healthcare utilization through admission avoidance, which will be reflected as an enhanced quality of life for the patient [100]. Although bodies such as the European Commission have highlighted the potential of telehealth in the management of chronic diseases, there is limited evidence for its effectiveness in COPD [101]. Although systematic reviews investigating the role of telehealth in patients with COPD have reported reductions in ED attendance and hospital admission [100,102,103], there has been a wide variation in the nature of the interventions themselves, with some of the studies being underpowered [102], such that clinical effectiveness has not been established. A meta-analysis reported that telemonitoring actually appeared to increase the mortality rate compared with UC, suggesting that patients may have delayed seeking urgent medical attention because of false reassurance from the remote assistance [103]. The Whole System Demonstrator study, a UK project funded by the Department of Health, was designed to establish whether integrated care supported by telehealthcare was effective in reducing healthcare utilization and mortality in a large number of patients with chronic illness, including COPD [104]. Primary care practices were randomized to provide either telehealth or UC, and patients were enrolled across three UK regions. Telehealth reduced the hospital admission and mortality [104], but interestingly, there was no improvement in either quality of life or psychological outcomes [105]. In a subsequent economic analysis, telehealth was not shown to be cost-effective in patients with COPD [106]. The results of a large UK RCT in patients with COPD are awaited [107]. In the interim, as part of a European Commission Innovation Partnership project, clinicians, researchers and engineers are working together identify the specific technological, physiological, behavioral, and clinical components that should be included in an advanced care coordination and telehealth deployment program to provide the most benefit to patients [108].

## Conclusions

Attention has been focused on the development of non-pharmacological strategies to improve health status and quality of life, and to reduce healthcare utilization and costs by preventing the frequency and severity of acute exacerbations of COPD. These non-pharmacological strategies, although they show potential, need further supporting data before widespread implementation can be suggested.

## Abbreviations

AHRF: Acute hypercapnic respiratory failure; COPD: Chronic obstructive pulmonary disease; CPAP: Continuous positive airway pressure; FEV1: Forced expiratory volume in 1 second; HRQL: Health-related quality of life; IL: Interleukin; IPAP: Inspiratory positive airway pressure; LTOT: Long-term oxygen therapy; NIV: Non-invasive ventilation; PR: Pulmonary rehabilitation; RCT: Randomised controlled trial; UC: Usual care.

## Competing interests

NH is in receipt of a European Union grant for the development of care coordination and telehealthcare for chronic diseases including COPD. ES is in receipt of an unrestricted educational grant from Philips Electronics to develop advanced physiological monitoring techniques in patients with COPD.

## Authors’ contributions

ES, SM, and NH contributed to the literature review and manuscript preparation. All authors have read and approved the final manuscript.
